# Teachers’ Burnout: The Role of Trait Emotional Intelligence and Social Support

**DOI:** 10.3389/fpsyg.2019.02743

**Published:** 2019-12-10

**Authors:** Caterina Fiorilli, Paula Benevene, Simona De Stasio, Ilaria Buonomo, Luciano Romano, Alessandro Pepe, Loredana Addimando

**Affiliations:** ^1^Department of Human Sciences, LUMSA University, Rome, Italy; ^2^Department of Human Science “R. Massa”, University of Milano-Bicocca, Milan, Italy; ^3^Department of Teaching and Learning, University of Applied Science and Arts of Southern Switzerland, Locarno, Switzerland

**Keywords:** burnout, trait emotional intelligence, social support, Italian teachers, family

## Abstract

The current study investigates the relations among teachers’ trait emotional intelligence, internal and external social support, and their levels of burnout. We hypothesized that both emotional intelligence and teachers’ perceived social support were associated with low level of teachers’ burnout. We further expected that internal and external support mediated the relationship between trait emotional intelligence and burnout scores. Participants were 318 in-service Italian teachers. The structural equation modeling analysis supports the idea that teachers’ trait emotional intelligence is strongly and directly associated with their burnout. Furthermore, internal social support (from the teachers’ workplace relationships) was more effective on burnout than support forthcoming from their external context. On the contrary, the mediation hypothesis was partially supported by the empirical data. These findings shed light on the relationship between teachers’ emotional competence and their burnout experience at school.

## Introduction

Trait Emotional Intelligence (trait EI) is a constellation of self-perceived, emotion-related abilities that enable individuals to recognize, process, and use emotional information (Sevdalis et al., 2007; Petrides, 2010). According to Petrides et al. (2016) trait EI refers to how people perceive their own emotional and social effectiveness, and represents a comprehensive dimension of the affective aspects of personality. It is generally operationalized in four components: general well-being (e.g., self-esteem, happiness, and optimism), self-control (e.g., stress management, and emotional control), emotionality (e.g., emotional perception and expression), and sociability (e.g., social awareness, emotional management, assertiveness, adaptability, and self-motivation).

Numerous studies have shown a strong association between trait EI and well-being in the workplace (e.g., Di Fabio and Kenny, 2016; Di Fabio, 2017). Individuals with high EI are more likely to see themselves as efficient, experience more positive than negative emotions, forge more positive relationships with others, and perceive everyday challenges in a way that promotes well-being, engagement, and job satisfaction (Maslach et al., 2001; Furnham and Petrides, 2003; Zeidner et al., 2004, 2012; Avsec et al., 2009; Brackett et al., 2010, 2011; Santisi et al., 2014; Benevene et al., 2018a, b). The literature on teaching has amply documented the role of trait EI in preventing adverse outcomes, such as burnout (O’Boyle et al., 2011; Mérida-López et al., 2017).

There is a wide agreement to consider burnout syndrome as a negative work-related outcome due to the long-term effect of strain resulting from repeated exposure to stressful events (e.g., Demerouti et al., 2002). According to several scholars the main core of burnout is feeling emotionally exhausted, which is characterized by physical and psychological fatigue (e.g., Travers, 2017) in three different social contexts, namely: private life, workplace, and relationship with clients (in this study with students) (Kristensen et al., 2005; Avanzi et al., 2014; Schonfeld et al., 2017). Furthermore, previous studies indicated that teachers’ burnout is also related to gender (i.e., females are more at risk than men) and positively associated with years of experience (i.e., high risk of burnout among less experienced teachers) (Leiter et al., 2014).

Teachers with more advanced emotional competencies are better equipped to handle the relative strain and emotional burden and to make sense of their reactions to sources of stress (e.g., Chan, 2006; Pishghadam and Sahebjam, 2012; Borrelli et al., 2014; Ramaci et al., 2016; Buonomo et al., 2017; De Stasio et al., 2017; Fiorilli et al., 2017a).

Moreover, teachers’ social support (internal and external to the workplace) may constitute a further resource when facing stressful events. Internal support comes from within the work setting itself (i.e., support from colleagues, supervisors, school leaders); whereas external support comes from the teachers’ private life (i.e., support from friends, family members, partners) (Schaufeli and Bakker, 2004; Gavish and Friedman, 2010; Carlson et al., 2014; McNall et al., 2015; Pérez-Fuentes et al., 2019). Internal support is generally associated with teachers’ sense of belonging, their commitment, and general well-being (e.g., Collie et al., 2017; Travers, 2017). On the other hand, external support has positive effects on job satisfaction and performance (Halbesleben, 2006; Skaalvik and Skaalvik, 2009; Carlson et al., 2014; McNall et al., 2015). Such findings come from a variety of cultural settings including Spain, Turkey, Korea, Jordan, Israel, Italy, and Palestine (Aycan and Eskin, 2005; Veronese et al., 2018; Fiorilli et al., 2019).

Overall, teachers’ social support seem to have a strong relationship with both teachers’ trait emotional intelligence, on one hand, and their burnout level, on the other. Due to the relevant implications of the trait EI in maintaining good social interactions (e.g., ability to interpret social cues, self-regulation of emotions, supportive social interactions), it is expected that teachers with high trait EI are more likely to positively perceive existing resources from both inside and outside their workplace (Gallagher and Vella-Brodrick, 2008).

Nevertheless, while the relationships among the aforementioned variables are well known and supported by substantial findings, the mediating role of teachers’ perception of their social support (both internal and external to the workplace) is less known. In one of the few studies that has investigated the mediating role of social support between trait emotional intelligence and burnout risk, the authors address the internal social support without any comparison with the external one (Ju et al., 2015).

Overall, an in-depth comparison of the two kinds of social support (internal versus external) is required to build up a more comprehensive picture of teachers’ burnout risk in relation to their personal characteristic named trait emotional intelligence.

### The Current Study: The Protective Role of Social Support

**FIGURE 1:**
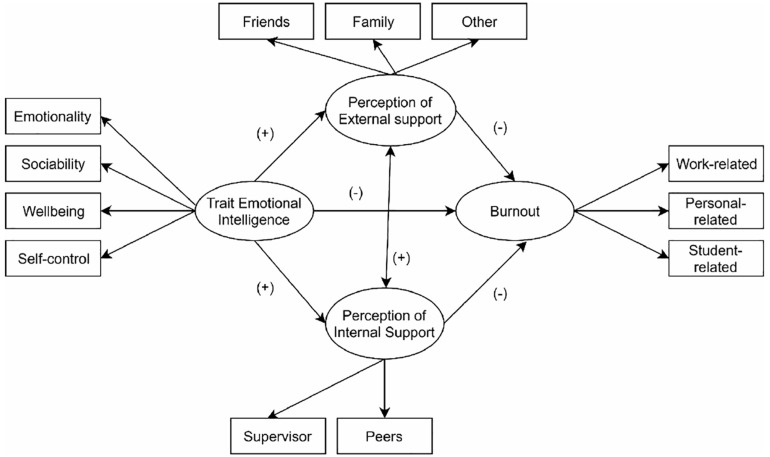
Hypothesized conceptual equation model of association among variables.

The aim current study was to model within a single structural equation model, the cumulative network of relationships among teachers’ trait emotional intelligence, perceived internal (e.g., colleagues and supervisor) and external social support (e.g., family and friends), and levels of professional burnout (see Figure 1). In line with evidences gathered in previous studies, the current study tested the following specific directional hypotheses: (1) teachers’ trait emotional intelligence would be negatively correlated with burnout scores as well as positively associated with perceived external and internal support. In turn, we also expected that both dimensions of social support (e.g., external and internal) were negatively associated with burnout scores; (2) the relationship between emotional trait intelligence and burnout scores would be mediate by perception of social support (e.g., external and internal). In order to test the network of associations among the variables of interest, structural equation modeling with the decomposition of total effects and analysis of regression coefficients was adopted (Byrne, 2013).

## Materials and Methods

### Participants

The sample was composed of 318 teachers (94.7% female) aged 27–65 years (*M* = 47.1, *SD* = 8.55) who were mostly employed in primary schools (71.2%) and public nursery schools (13.1%) in Northern (8.8%), Central (66.1%), and Southern Italy (25.1%). Most of the participants were tenured teachers (72.3%); about 63% held a master’s degree or other postgraduate qualifications, and 37% held a high school diploma. Their years of teaching experience ranged from 1 to 41 (*M* = 18.1, *SD* = 11.1).

### Procedure

The data was collected in late 2018 in seven schools in Northern, Central, and Southern Italy. Having obtained approval from the school principals, a researcher contacted the teachers informing them about the study and inviting them to participate. Paper and pencil questionnaires were used for data collection. All participants signed informed consent forms and were informed about the anonymity and confidentiality of their answers. The research protocol was approved by the Ethics Committee of the LUMSA University, Rome.

### Instruments

#### Trait Emotional Intelligence Questionnaire (TEIQ)

*Trait Emotional Intelligence* was assessed via the Italian-language version of the 30-item Trait Emotional Intelligence Questionnaire-Short Form for Adults (TEIQue-ASF; Petrides, 2009; Italian validation by Di Fabio, 2013). The TEIQue-ASF is a self-report questionnaire whose items (e.g., “Expressing my emotions with words is not a problem for me”; “I often find it difficult to see things from another person’s viewpoint”) are rated on a 7-point Likert scale, ranging from 1 (completely disagree) to 7 (completely agree). The Italian version of the TEIQue-ASF measures four latent factors: Emotionality (eight items), Sociability (five items), Self-control (seven items), and Well-being (10 items). In the current study, the alpha coefficients ranged from 0.716 to 0.658, whereas the overall reliability for the measure was equal to 0.868.

#### Internal Social Support (HSE)

We used the published Italian version of the *Health and Safety Executive Stress Indicator Tool* (Balducci et al., 2015) regarding teachers’ perception of social support coming from the school network to deal with stressful events. In the current study we used two subscales, namely: Supervisor Support (five items) and Peer Support (four items). Examples of items in these subscales included the following: “I am given supportive feedback on the work I do” (for Supervisor Support), and “If work gets difficult, my colleagues will help me” (for Peer Support). All items were answered by using a Likert-Type response scale ranging from 1 (Never) to 5 (Always). In the current study the alpha reliability coefficients were 0.893 (peer support) to 0.904 (supervisor support).

#### External Social Support (MSPSS)

The *Multidimensional Scale of Perceived Social Support* (Zimet et al., 1988; Di Fabio and Busoni, 2008) is an Italian 12-item self-report questionnaire focused on teachers’ perception of social support coming from their private life and able to support them in school-related stressful events. A few examples of the items included the following: Significant Other (four items) (e.g., “There is a special person who is around when I am in need”), Family (four items) (e.g., “My family really tries to help me”), and Friends (four items) (e.g., “I have friends with whom I can share my joys and sorrows”). Items were scored by using a Likert-Type response scale ranging from 1 (Very Strongly disagree) to 7 (Very strongly Agree). In the current study the alpha coefficients ranged from 0.930 (family) to 0.870 (significant others).

#### Burnout

We used the published Italian version of the *Copenhagen Burnout Inventory* (Kristensen et al., 2005; Fiorilli et al., 2015). This self-report questionnaire is composed of three subscales with a total of 19 items, each rated on a 5-point Likert scale. The three subscales are: Personal Burnout (six items) (e.g., “How often do you feel tired?”), Work-related Burnout (seven items) (e.g., “Do you feel worn out at the end of the working day?”), and Student-related Burnout (6 items) (e.g., “Do you find it hard to work with students?”). Items were scored by using a Likert-Type response scale ranging from 1 (Never) to 5 (Always). In the current study, the alpha coefficients were as follows: personal burnout (0.886), work-related burnout (0.875), and student-related burnout (0.846).

### Analytic Strategy and Data Modeling

Data analytic strategy consisted of two sequential stages. First, main descriptive statistics, as well as zero-order correlations, were computed. The presence of multivariate outliers was assessed by computing Mahalanobis’ distance (*p* < 0.001) for all variables. No multivariate extreme values were found and consequently removed from the sample. The distribution of empirical indicators was then explored. None of the variables under study reported kurtosis or skewness values falling outside the recommended thresholds of +2 and −2 (George and Mallery, 2010), and they resembled a normal distribution.

As required in the case of structural equation modeling strategies (Kline, 2011; Pepe et al., 2017, 2018; Veronese et al., 2017), the goodness of fit indexes were analyzed to estimate the overlap between the observed matrix of covariances (S) and the reproduced matrix of covariances (Σ). Thresholds for good model fit were: RMSEA < 0.07 (Schermelleh-Engel et al., 2003), NFI > 0.95, NNFI > 0.95 (Marsh et al., 2004; Nagengast et al., 2014), CFI > 0.95 (Hu and Bentler, 1999). The maximum likelihood method (Kline, 2011) was used to estimate the parameters for the structural models. All analyses were conducted by using SPSS AMOS 23 (Arbuckle, 2014).

## Results

The results are presented in two sections covering general descriptive statistics/zero-correlations and the results of the structural model. Descriptive statistics and zero-order correlations are summarized in Table 1.

**TABLE 1:** Descriptive statistics and zero-order correlations of trait emotional intelligence, internal support, external support, and burnout (*N* = 318).

	**1**	**2**	**3**	**4**	**5**	**6**	**7**	**8**	**9**	**10**	**11**	**12**	**13**	**14**
(1) Emotionality (TEI)	–													
(2) Sociability (TEI)	0.399^∗∗^	–												
(3) Well-being (TEI)	0.434^∗∗^	0.540^∗∗^	–											
(4) Self-control (TEI)	0.458^∗∗^	0.442^∗∗^	0.520^∗∗^	–										
(5) Supervisor support (HSE)	0.173^∗∗^	0.275^∗∗^	0.237^∗∗^	0.160^∗∗^	–									
(6) Peer support (HSE)	0.153^∗∗^	0.291^∗∗^	0.212^∗∗^	0.182^∗∗^	0.657^∗∗^	–								
(7) Family subscale (MSPSS)	0.175^∗∗^	0.159^∗∗^	0.187^∗∗^	0.150^∗∗^	0.180^∗∗^	0.183^∗∗^	–							
(8) Friends subscale (MSPSS)	0.210^∗∗^	0.187^∗∗^	0.261^∗∗^	0.197^∗∗^	0.282^∗∗^	0.340^∗∗^	0.467^∗∗^	–						
(9) Significant others (MSPSS)	0.108	0.095	0.093	0.031	0.277^∗∗^	0.308^∗∗^	0.454^∗∗^	0.573^∗∗^	–					
(10) Personal burnout (CBI-IT)	–0.196^∗∗^	–0.299^∗∗^	–0.435^∗∗^	–0.380^∗∗^	−0.112^∗^	–0.149^∗∗^	−0.126^∗^	–0.090	0.033	–				
(11) Work-related burnout (CBI-IT)	–0.284^∗∗^	–0.398^∗∗^	–0.460^∗∗^	–0.461^∗∗^	–0.233^∗∗^	–0.261^∗∗^	–0.208^∗∗^	−0.134^∗^	–0.022	0.784^∗∗^	–			
(12) Student-related burnout (CBI-IT)	–0.264^∗∗^	–0.342^∗∗^	–0.372^∗∗^	–0.353^∗∗^	–0.102	−0.114^∗^	–0.185^∗∗^	−0.141^∗^	–0.087	0.468^∗∗^	0.614^∗∗^	–		
(13) Working experience (years)	–0.064	–0.084	–0.079	0.011	−0.116^∗^	–0.085	−0.113^∗^	–0.016	–0.049	0.030	0.101	0.244^∗∗^	–	
(14) Gender	−0.142^∗^	–0.088	–0.145^∗∗^	–0.035	–0.175^∗∗^	–0.178^∗∗^	–0.160^∗∗^	–0.108	–0.066	0.031	0.089	0.192^∗∗^	0.830^∗∗^	–
Mean	29.32	19.69	3.30	3.87	16.63	14.86	18.97	18.19	18.22	16.41	16.57	12.31	18.10	–
Standard deviation	4.76	3.47	4.48	5.69	4.35	3.10	4.12	3.63	4.09	4.50	5.36	4.55	11.12	–
Skewness	–0.463	–0.078	–1.017	–0.053	–0.169	–0.337	–1.427	–1.090	–1.200	0.198	0.488	0.849	–1.139	–

*^∗^*p* < 0.05; ^∗∗^*p* < 0.01; Male (gender = 0) was used as baseline reference. TEI, Trait Emotional Intelligence; HSE, Internal support measures; MSPSS, External support measures; CBI-IT, Copenhagen Burnout Inventory.*

**FIGURE 2:**
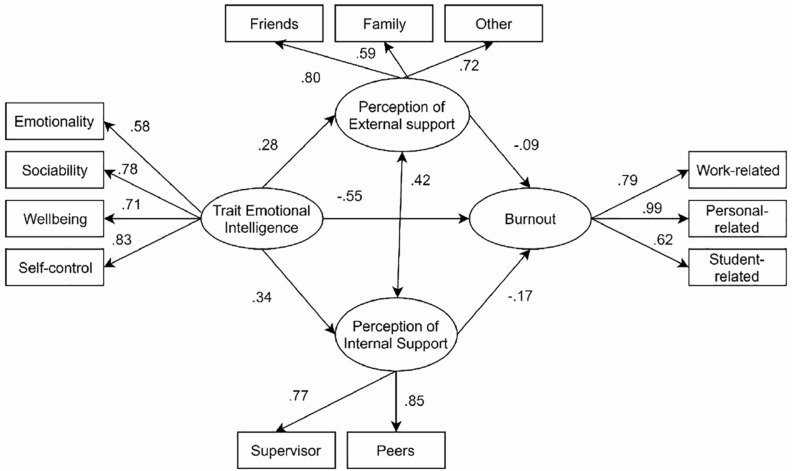
Results of the conceptual equation model, standardized direct effect were reported.

Trait emotional intelligence and burnout scores showed negative medium-large and statistically significant correlations. In particular, well-being scores were negatively correlated to work-related burnout (*r* = −0.460, *p* < 0.001) and personal-related burnout (*r* = −0.435, *p* < 0.001). Similarly, negative correlations were found between student-related burnout and both sociability (*r* = −0.342, *p* < 0.001), and self-control (*r* = −0.353, *p* < 0.001). Besides, work-related burnout scores were generally correlated negatively with all TEIQ subscales. Moreover, trait emotional intelligence was positively associated with internal as well as external support that teachers perceived by their sources. With regard to the associations between burnout and social support, we found that, as expected, negative correlations with all subscales, with the exception of the student-related burnout that showed no association with supervisor support. The results of the structural equation model are reported in Figure 2.

The analysis of goodness of fit indexes supported the acceptance of the proposed conceptual model (χ^2^(58) = 120.3, *p* < 0.01; NC = 2.07; RMSEA = 0.058, CI 90% [0.043;0.073], NFI = 0.928, NNFI = 0.961, CFI = 0.961), suggesting that the effects among the modeled variables were both conceptually and statistically significant. The main paths of the structural model were then assessed by decomposing the total standardized effects in direct and indirect effects (standardized values are reported in Figure 2), in order to identify the directions and the sizes of the associations. With regard to the second hypothesis, which focused on the direct effects among studied variables, we found a negative total effect of teachers’ trait EI on burnout levels (β = −0.63, *p* = 0.010). Whereas trait EI resulted in two positive direct effects, similar in both size and direction, with perceptions of external support (β = 0.28, *p* = 0.008) and internal support (β = 0.34, *p* = 0.010). Finally, two others direct negative associations were estimated in relation to burnout scores, with both internal (β = −0.17, *p* = 0.007) and external (β = −0.09, *p* = 0.029) support reporting negative direct effects, meaning that the higher the perceptions of being supported (i.e., internally and externally) the lower the burnout levels (see Table 2 for details).

**TABLE 2:** Summary of direct, indirect, and total standardized effect.

**Parameters**		**Standardized coefficient**					
**From**	**To**	**Direct**	**95th CI**	**Indirect**	**95th CI**	**Total**	**95th CI**
Trait emotional intelligence	Emotional support	0.344^∗∗^	0.046 to 0.111	N/A	N/A	0.344^∗∗^	0.046 to 0.111
Trait emotional intelligence	Internal support	0.278^∗∗^	0.155 to 0.429	N/A	N/A	0.278^∗∗^	0.155 to 0.429
Trait emotional intelligence	Burnout	–0.553^∗∗^	−0.875 to −0.516	−0.081^∗^	−0.10 to 0.007	–0.634^∗∗^	−0.921 to −0.600
Emotional support	Burnout	–0.165^∗∗^	−1.738 to −0.452	N/A	N/A	–0.165^∗∗^	−1.738 to −0.452
Internal support	Burnout	−0.089^∗^	−0.036 to 0.002	N/A	N/A	−0.089^∗^	−0.036 to 0.002

*N/A, not applicable; 95th CI = confidence intervals, ^∗^*p* < 0.05, ^∗∗^*p* < 0.01. Indirect effect of trait emotional intelligence on burnout (−0.083) was the sum of standardized indirect effects via emotional support (−0.024^∗^, 95th CI [−1.139 to −0.043]) and internal support (−0.057^∗^, 95th CI [−1.358 to −0.059]).*

Regarding the indirect effects of social support perceived by teachers, we found a very minimal mediating role of teachers’ social support (both internal and external) on the relationship between trait EI and burnout levels. A closer look into the composition of the total effect (β = −0.63, *p* = 0.010) in direct (β = −0.55, *p* = 0.016) and indirect (β = −0.08, *p* = 0.028) components revealed that the effect of emotional intelligence trait on burnout (via different type of social support) was only minimal.

## Discussion

In this study, we investigated the role played by perceived social support in the relationship between teachers’ trait emotional intelligence and levels of burnout. As predicted, all the key research variables were significantly associated showing that high level of teachers’ trait emotional intelligence (i.e., emotionality, sociability, well-being, and self-control) was associated with low burnout level (i.e., personal, work-related, and student-related). As expected, and in line with previous research (e.g., O’Boyle et al., 2011; Schutte and Loi, 2014), teachers’ trait EI gives them emotional resources to face school-related stressful events (i.e., work-related burnout and student-related burnout) as well as stressful events in their own private life (i.e., personal burnout). Coherently with the personality-based dimension approach (Petrides et al., 2016), trait EI is a dimension able to predict a wide range of people’s well-being (e.g., high level of happiness, well-being, and satisfactory interpersonal relationships) by having an impact on several life contexts (e.g., Vergara et al., 2015). In the same direction we found that the more social support (from both inside and outside the school setting) the teachers perceived, the less they experienced burnout. According to previous research (Fiorilli et al., 2017a), when teachers show high satisfaction with support perceived as available to them their burnout risk decreases. Surprisingly, no association was found between student-related burnout and support coming from teachers’ supervisors. Previous findings have shown that supervisors are one of the most important support mechanisms for teachers who may feel more effective in their work with students by having a greater sense of autonomy. However, the role played by supervisors is related, among others variables, with teachers’ years of experience that, in the current study, has shown a very weak association. This leads us to believe that further investigations with a larger sample of teachers may be needed on this association (e.g., Van Droogenbroeck et al., 2014).

Moreover, in line with previous findings (Halbesleben, 2006; Pomaki et al., 2010; Pietarinen et al., 2013), we found a strong association between teachers’ personal resources (in this case, trait EI) and social support. In practice, teachers with high trait EI perceived as effective the support coming from internal and external sources. This was an expected finding, given the existing literature addressing the relationships between peoples’ trait EI and their positive attitude toward coping strategies, such as seeking support to face difficult situations (Andrei and Petrides, 2013). Furthermore, no associations were found between all subscales of TEIQ and the additionally external social support coming from significant others. It is an unexpected result that may lead to reconsider the informative value of the subscale labeled “significant others” within the same instrument where family and friends dimensions may exhaustively explore peoples’ relations. In this regard, it has to be stressed that the Multidimensional Scale of Perceived Social Support was administered in the Italian context only among students (Di Fabio, 2015). Whereas in the current study we addressed workplace life by asking teachers about the sources available to support them in school-related stressful events. In this regard, friends’ support subscale may overlap with the significant other subscale.

With regard to the direct and indirect relationship among studied variables, the findings partially supported our hypotheses. First, teachers’ trait emotional intelligence may buffer individuals from burnout and makes them more inclined toward receiving social support from internal as well as external sources. Second, comparison of the direct and indirect effects of trait emotional intelligence on levels of burnout in our sample of teachers suggests that the indirect effect of trait EI, via social support, is trivial if compared to its direct effect. Looking at the trait EI dimensions we can observe that high level in sociability, emotionality, well-being, and self-control gives people a set of several competences to face with emotional events where it is presumable that perceived social support plays an important role, but not essential, as our findings seem to support. The direct impact of trait EI on teacher burnout identified in this study is characterized by an extensive body of research suggesting that burnout is a subjective phenomenon that varies as a function of the interaction between personal resources and events experienced (Lambert et al., 2009; Platsidou, 2010; Fiorilli et al., 2017b). The components of trait EI include general well-being, sociability and self-control, all of which may reasonably be expected to help teachers adopt a positive perspective on critical school events (Miao et al., 2016). This appears to be particularly true in light of the weak indirect effect – via social support – of trait EI on burnout in our sample. In other words, teachers’ trait emotional intelligence gives them sufficient resources in terms of stress management, empathy, and social awareness that may assure them adaptability to workplace challenges (Petrides et al., 2016). More specifically, teachers’ trait EI leads teachers to be less vulnerable to burnout by giving them more sensible and effective ways to use emotional information coming from their workplace context (Brackett et al., 2010), as well as their personal life.

Furthermore, we found that internal social support reported a higher effect on burnout levels than support received from the teachers’ private life. More specifically, teachers with high trait EI are more likely to positively perceive the support coming from their supervisor and colleagues than from their private life. A perspective focuses on workplace well-being leads us to positively evaluate teachers’ confidence in internal social support. Effectively, colleagues and supervisors may be more likely to support workers by helping them in solving problems and implementing new professional strategies and practices. Even though these results require further investigation, by taking into account the role played by all subscales of external support in explaining its role (see the not significant association found in relation to significant others subscale which may partially account for the weaker role of external support compared with the internal one), it is important to highlight the extensive literature supporting the relevant role of the supervisor and colleagues in helping teachers with their school-life challenges (Day and Leiter, 2014).

## Conclusion, Limitations, and Future Research Directions

Theoretical perspectives on trait EI suggest routes to enhancing teachers’ personal resources and thereby their ability to cope with stressful events. More specifically our findings suggest that personal competence in the emotional domain may significantly contribute in teachers’ well-being rather than social support itself. Importantly, trait EI has proved susceptible to improvement through training programs (Vesely et al., 2014), and improved EI – as the present findings confirm – can help to prevent teacher burnout (Brackett and Katulak, 2006). Our findings lead us to take into account the implications for teachers’ professional development. Despite the fact that trait EI is a personal variable, it is not unalterable one. On the contrary, it is susceptible to improvement through training programs (Brackett and Katulak, 2006; Vesely et al., 2014). Additionally, in line with previous research (e.g., Demerouti, 2014), our results should seriously lead us to consider individual strategies as key variables for reducing burnout as well as enhancing personal resources. In the current study, self-control is the most relevant dimension within teachers’ trait EI that may support the idea that their ability to manage emotions in the social interactions could be a guideline for the teachers’ professional development programs.

The current study displayed some limitations that should be taken into account in future research. First, longitudinal studies are required to establish whether internal and external support available to teachers result and/or impact their trait emotional intelligence. Second, future research should include an analysis of contextual factors such as leadership from the school principal and school climate (e.g., McCarthy et al., 2017) in order to further understand how teachers’ evaluation of internal support may depend on the quality of workplace resources. Third, in future research, both the quality and quantity of teachers’ relationships with other teachers should be assessed in greater depth and using a range of measures, rather than exclusively relying on self-report instruments as in the present study. More specifically, using a qualitative method for collecting data (e.g., interviews and observations) may shed a light on the quality of each social support (i.e., whether it offers emotional support, instrumental or informative support).

## Data Availability Statement

The datasets for this article are not publicly available because of local legal and privacy restrictions (Italian Data Protection Code – Legislative Decree No. 196/2003).

## Ethics Statement

The authors assert that all procedures contributing to this work comply with the ethical standards of the relevant national and institutional committees on human experimentation. All participants gave written informed consent in accordance with the Declaration of Helsinki.

## Author Contributions

CF, IB, and LR designed and carried out the study and contributed to the analysis of the results and writing of the manuscript. AP collected data and contributed to the analysis of the results and writing of the manuscript. PB, SD, and LA supervised the study design and the manuscript draft.

## Conflict of Interest

The authors declare that the research was conducted in the absence of any commercial or financial relationships that could be construed as a potential conflict of interest.
